# A transposable element prevents severe hemophilia B and provides insights into the evolution of new- and old world primates

**DOI:** 10.1371/journal.pone.0312303

**Published:** 2024-10-18

**Authors:** Johannes Kopp, Alice Rovai, Michael Ott, Heiner Wedemeyer, Andreas Tiede, Hans Jürgen Böhmer, Tomas Marques, Jörg Langemeier, Jens Bohne, Simon Alexander Krooss

**Affiliations:** 1 Institute of Medical Genetics and Human Genetics, Charité –Universitätsmedizin Berlin, Freie Universität Berlin and Humboldt-Universität zu Berlin, Berlin, Germany; 2 Max Planck Institute for Molecular Genetics, RG Development & Disease, Berlin, Germany; 3 Department of Biology, Chemistry and Pharmacy, Institute of Chemistry and Biochemistry, Freie Universität Berlin, Berlin, Germany; 4 Institute of Transfusion Medicine and Transplant Engineering, Hannover Medical School, Hannover, Germany; 5 Department of Gastroenterology, Hepatology, Infection Diseases and Endocrinology, Hannover Medical School, Hannover, Germany; 6 Clinic for Hematology, Hemostaseology, Oncology and Stem Cell Transplantation, Hannover Medical School, Hannover, Germany; 7 Institute of Geobotany, Leibniz University Hannover, Hannover, Germany; 8 Institut Biologica Evolutiva, Universitat Pompeu Fabra, Barcelona, Spain; 9 Institute of Virology, Hannover Medical School, Hannover, Germany; Shandong Agricultural University, CHINA

## Abstract

Alu-elements comprise a large part of the human genome and some insertions have been shown to cause diseases. Here, we illuminate the protective role of an Alu-element in the 3’UTR of the human Factor 9 gene and its ability to ameliorate a poly(A) site mutation in a hemophilia B patient, preventing him from developing a severe disease. Using a minigene, we examined the disease-causing mutation and the modifying effect of the transposon *in cellulo*. Further, we simulated evolutionary scenarios regarding alternative polyadenylation before and after Alu insertion. A sequence analysis revealed that Old World monkeys displayed a highly conserved polyadenylation sites in this Alu-element, whereas New World monkeys lacked this motif, indicating a selective pressure. We conclude that this transposon has inserted shortly before the separation of Old and New World monkeys and thus also serves as a molecular landmark in primate evolution.

## Introduction

Hemophilia B is a clotting disorder caused by mutations in the human coagulation factor IX gene (hF9), which codes for a serine protease in the intrinsic pathway of the coagulation cascade. With a prevalence of approximately 1:30.000 in males, it represents an orphan disease. The factor IX variant database (www.factorix.org) currently lists 1692 variants (August 2023) in coding and non-coding regions of the F9 gene. The F9 gene is located on the X-chromosome (Xq27.1-q27.2).

The maturation of eukaryotic mRNA involves various steps such as 5’-capping, splicing and polyadenylation, which facilitate nuclear export and protection from exonucleases [1,2]. mRNAs without a poly(A) tail are degraded rapidly by exonucleases [3]. The process of polyadenylation is initiated by recognition of the hexameric poly(A) signal (PAS) AATAAA by the cleavage and polyadenylation specificity factor CPSF [4]. In addition to the binding of CPSF to the PAS, a G/U-rich sequence downstream named downstream sequence element (DSE) is recognized by the Cleavage stimulating Factor (CstF) [5]. Subsequently, the endonuclease CPSF73 together with other key players such as Cleavage Factors I and II (CF I, CF II) cut the RNA molecule approximately 10–30 nucleotides downstream of the PAS at the Cleavage Site (CS), which is normally composed of a UA or CA dinucleotide [6], where the poly(A) polymerase (PAP) binds and starts operating and adds around 250 Adenine-nucleotide residues [4,7]. The poly(A) tail is then recognized and bound by poly(A) binding proteins (PABP), which act as regulators of translation [8]. Besides the consensus motif of AATAAA alternative PAS such as ATTAAA or AATGAA do exist but exhibit a reduced polyadenylation- and cleavage activity as systematically demonstrated by Sheets and colleagues [9]. Therefore, mutations in the PAS can significantly reduce polyadenylation efficiency and impede gene expression.

More than two thirds of human genes show alternative polyadenylation (APA), which offers the possibility of generating different transcript isoforms. The usage of alternative PAS modulates the length and thus the properties of a 3’UTR as microRNA binding sites and secondary structures are located within these sequences [10,11]. The modulation of 3’UTR length has been shown to be of importance during physiological processes such as embryonic development where prolonged 3’UTRs were observed [12] and in T-cell activation, where shortened 3’UTRs were associated with enhanced growth advantages [13]. But also pathological processes involve APA during oncogene activation [14–16].

Alu elements are primate specific transposable elements with a length of approximately 300 bp. They belong to the group of short interspersed nuclear elements (SINEs). With more than one million copies, they make up more than 10% of the human genome [17]. Approximately 65 million years ago (mya) Alu elements have developed from a duplication of the 7SL-RNA gene, which is a component of the signal recognition particle [18–20]. Alu elements can be subdivided into various subfamilies of which the Alu J subfamily represents the earliest one followed by the Alu S subfamily, which includes *Sx*, *Sq*, *Sp* and *Sc* types. Alu Y elements represent the youngest subfamily largely abundant in old world monkeys and humans [21].

The insertion of Alu elements has been shown to cause various genetic defects such as hemophilia A, X-SCID, breast cancer or lipoprotein lipase deficiency [22]. The major pathological mechanism, however, remains *de novo* insertion leading to either disruption of open reading frames, splice sites or regulatory sequences [22]. Very recently, Alu elements have been shown to be involved in promoter-enhancer wiring via RNA sequence interactions [23]. Notably, the younger Alu-elements of the Y-subfamily appear to be predominantly associated with genetic diseases. In general, Alu elements have a terminal A-stretch, which can accumulate mutations or shorten over time, reflecting their transposition activity [24]. Therefore, a simple A>T transversion can directly lead to the generation of a PAS (AATAAA) within the terminal A-stretch. So far, only disease-causing effects through premature transcriptional termination were reported [25]. Here, we describe a hemophilia B patient, who apparently profits from the presence of an Alu *Sx* element harboring three functional PAS, as they appear to substitute the function of the mutated authentic F9 PAS1.

## Materials and methods

### Cell culture

HEK 293T-Cells are a Human embryonic kidney cell line, which expresses the Simian-Virus 40 (SV40) large T-Antigen. Cells were cultured in T-75 flasks at 37°C and 5% CO_2_ in a sterile incubator. Passaging occurred every 2–3 days when the cells reached a confluency of >90%. Cells were washed with PBS and treated with 1 mL of trypsin solution. After 2–3 minutes of incubation, the cells were carefully removed from the flask surface and split in a 1:10 manner. Subsequently, fresh DMEM media supplemented with 1% Penicillin/Streptomycin, 10% FCS and 1% sodium pyruvate was added and the flask was gently swirled to equally distribute the cells. Transfection was performed according to the manufacturer’s protocol of Lipofectamine 3000 (Invitrogen).

Half-life experiments were conducted in HeLa TA cells harboring a stably integrated copy of the Tet-transactivator by the addition of 20 ng Doxycyclin.

For FIX activity analysis, 4×10^5 HEK 293T cells were seeded in a six-well. At a confluency of about 50%, cells were transfected with various FIX minigenes using Viafect (Promega). 12 hours post transfection, medium was replaced with FCS-free DMEM, supplemented with 4μg/mL vitamin K, 2.5% BSA and 1% L-Glutamine and cells were incubated for 96 hours.

### Cloning work

The generation of pSK_SV40_F9_wt is described in Krooss et al. 2020. To introduce an alternative poly(A) signal inactivating mutation, the source plasmid pSK_SV40_F9_wt was modified via PCR mutagenesis in the following way: Using the forward primer P1, which includes an XbaI site 5’ (underlined) and the according modification of the poly(A) signal (bold), and the reverse primer P4 a PCR was performed on the SV40_F9_wt template. The PCR-product was then digested using XbaI/MunI and ligated into the linearized target plasmid, thereby giving rise to pSK_SV40_F9_PAS1mut2. For the generation of the PAS1 mutation as described in Li et al. 2000, the SV40-driven minigene published in Krooss et al. 2020 was modified via PCR mutagenesis in the following way similar to pSK_SV40_F9_PASmut1: Using the forward primer P2, which includes an XbaI site 5’ (underlined) and the according modification of the poly(A) signal (bold), and the reverse primer P4 a PCR was performed on the SV40_F9_wt template. The PCR-product was then digested using XbaI/MunI and ligated into the linearized target plasmid, thereby giving rise to pSK_SV40_F9_PAS1mut. For the generation of another alteration of the PAS1, the SV40-driven minigene published in Krooss et al. 2020 was modified via PCR mutagenesis in the following way similar to pSK_SV40_F9_PASmut1 and pSK_SV40_F9_PAS2mut: Using the forward primer P3, which includes an XbaI site 5’ (underlined) and the according modification of the poly(A) signal (bold), and the reverse primer P4 a PCR was performed on the SV40_F9_wt template. The PCR-product was then digested using XbaI/MunI and ligated into the linearized target plasmid, thereby giving rise to pSK_SV40_F9_PAS1mut. The deletion of the Alu *Sx* element from the source plasmid pSK_SV40_F9_wt was performed via overlap PCR using primers P5+P6 and P7+P8 and a fusion of the products in a final PCR using P6+P8 with subsequent cloning via EcoRI/XbaI. The canine 3’UTR was extracted from Madin-Darby canine kidney (MDCK) cells using primers P13 and P14. Subsequently the PCR product and the target vector (pSK_SV40_F9_wt) were digested using BamHI/MunI and ligation was performed.

To generate the shut off vectors, F9 plasmids pSK_SV40_F9_wt and pSK_SV40_F9_PAS1mut were amplified using P16+P17 and transferred into a pTetbi promoter driven vector (kindly provided by V. Cordes, MPI Göttingen) via AgeI, BamHI and NcoI digest.

### RNA work

RNA methods were performed as described previously [26]. For detection of F9 RNA, a specific probe was generated from the FIX plasmid by HindIII/BamHI digestion. The GAPDH-specific probe was prepared as described. cDNA synthesis for quantitative PCR (qPCR) was conducted using QuantiTect Reverse Transcription Kit (Qiagen) according to the manufacturer’s protocol. Prior to reverse transcription, RNA was treated with TURBO DNase (Invitrogen) and purified via RNeasy columns (Qiagen). qPCR was performed with QuantiTect SYBR Green PCR Kit (Qiagen) using primer pairs P9+P10 (F9) and P11+P12 (GAPDH). For 3’RACE experiments, poly(A)+ -RNA of transfected cells was isolated and 50 ng were reverse transcribed. To this, the Invitrogen 3’-RACE protocol and the provided reagents were utilized. The PCR products were separated via agarose gel electrophoresis. After gel extraction, the DNA was transferred into pCR2.1 (Invitrogen) for sequencing. Northern blot using total RNA obtained from HEK 293T cells transfected with the indicated constructs. The membrane was hybridized with a P32-labelled probe corresponding to the F9 cDNA. The position of F9 mRNA is indicated on the right. Glyceraldehyde 3-phosphate dehydrogenase (GAPDH) serves as a loading control. Bands were densitometrically quantified using a phosphoimager.

#### Factor IX activity measurement

FIX activity was determined as described in Krooss et al. 2020 using a one-stage coagulation assay. Samples were diluted 1:5 in imidazol buffer and mixed with an equal amount of FIX deficient plasma and Actin FS APTT reagent (all from Siemens Healthcare). After incubation at 37°C for 2 min, 0.025 M calcium-chloride was added, and the coagulation time recorded in an Amelung KC10 coagulometer. Calibration curves were generated with human standard plasma diluted in FIX deficient plasma.

## Results

### A Patient with a severe poly(A) site mutation displays regular F9 mRNA levels

**Fig 1:**
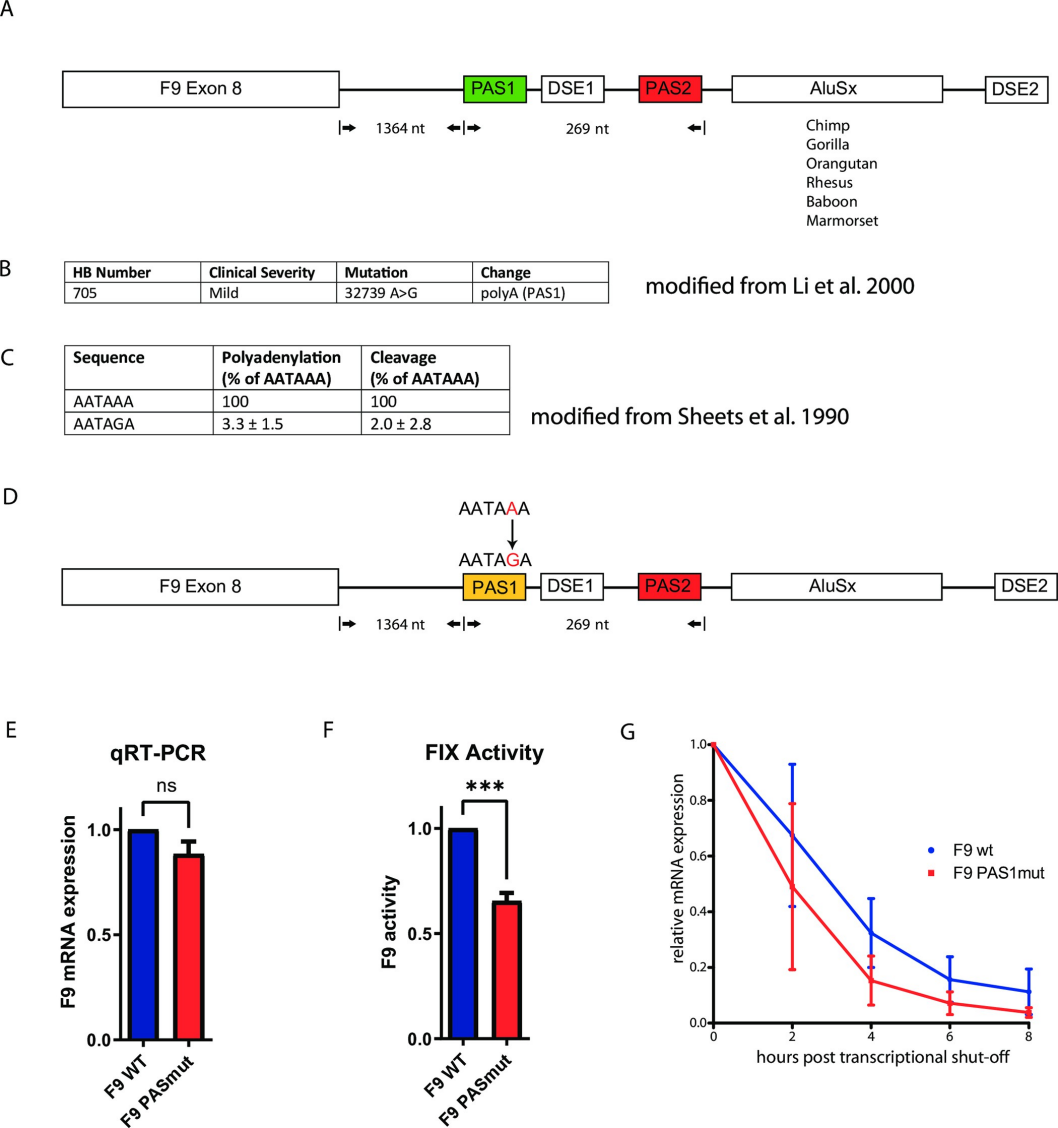
A predicted devastating mutation in the F9 PAS only causes a mild hemophilia B. **A)** Architecture of the F9 3’UTR and downstream sequence. PAS1 is located 1364 nucleotides downstream of F9 exon 8. It is followed by its authentic DSE and another PAS (PAS2), which has been shown to be inactive (marked in red). 3’ of PAS2 the Alu Sx element is located and interspaces PAS2 from its putative DSE (DSE2). **B)** Description of the pathogenic mutation including the nucleotide position (modified from Li et al. 2000(28)). **C)** Depiction of polyadenylation- and cleavage activity of a standard poly(A) signal and the mutated one in the patient. The activity of these modifications was measured in the context of ß-globin mRNA by Sheets et al. (1990). **D)** Depiction of the F9 minigene holding the patient’s mutation in the 3’UTR. **E)** qRT-PCR on F9 mRNA upon transfection of the F9 minigenes into HEK 293T cells, normalized to GAPDH (n = 3 biological replicates, error bars represent standard deviation. Students t test; ns: not significant.). **F)** FIX clotting activity measured by a one-step clotting assay in supernatant obtained from F9 minigene-transfected HEK 293T cells (n = 3 biological replicates, bars represent standard deviation, unpaired two-tailed t test; p = 0.0008). **G)** Measurement of F9 mRNA half-life using an expression shut-off vector. pTetbi F9wt and pTetbi F9_PAS1mut plasmids were transfected into Hela TA cells. 36 hours post transfection, doxycycline was added to shut off transcription. mRNA was purified at several timepoints after transcriptional shut-off. mRNA amounts were determined via Northern, bars represent standard deviation).

The human F9 3’UTR incorporates one actual PAS and one cryptic PAS, which are interspaced by 210 nucleotides. Earlier, we have demonstrated that physiologically only the first poly(A) signal (PAS1) is utilized for hF9 mRNA polyadenylation [26]. Initial polyadenylation analysis of the factor IX mRNA revealed exclusive utilization of PAS1 and no evidence for PAS2 activity as determined by 3’RACE (S1 Fig in S1 File). The inactivity of PAS2 can be explained by its increased distance to DSE2 as a consequence of the Alu *Sx* element insertion, since the physiological distance between PAS and DSE does not exceed more than approximately 60 bp [27]. This Alu element is conserved in various species as depicted in Fig 1A.

In 2000 Li et al. [28] have described a hemophilia B patient with a poly(A) site mutation (32739 A>G) in the F9 gene (Fig 1B). However, the disease severity of this individual was classified as mild, which intriguingly does not reflect the expected mRNA expression level, assuming that an AATAAA>AATAGA conversion leads to a residual polyadenylation activity of approximately 3% as demonstrated by Sheets et al. [9] (Fig 1C). The weak predicted residual activity of the mutated poly(A) site, together with our earlier finding that exclusively PAS1 appears to be functional (S1 Fig in S1 File), encouraged us to modify our previously established F9 minigene accordingly to mimic the genomic situation of this patient (Fig 1D). Despite the predicted polyadenylation efficiency of 3%, F9 mRNA levels determined via RT-qPCR revealed only a slight decrease of mRNA amounts when compared to healthy donor F9 mRNA (Fig 1E). However, significant differences in coagulation activity (secreted Factor IX) were detected upon analyzing the media of the transfected cells (Fig 1F). To test, whether a different mRNA half-life would be responsible for the reduced activity on protein level, we constructed inducible vectors that can facilitate transcriptional shut-off immediately upon doxycycline exposition. Total mRNA was harvested 2, 4, 6, and 8 hours post induction and F9 specific mRNA was determined. The results of the half-life analysis indicate a reduced half-life for the patient’s F9 mRNA when compared to healthy donor control (Fig 1G), despite some variation at the 2 h time point.

### An Alu-element protects the patient’s mRNA by facilitating alternative polyadenylation

**Fig 2:**
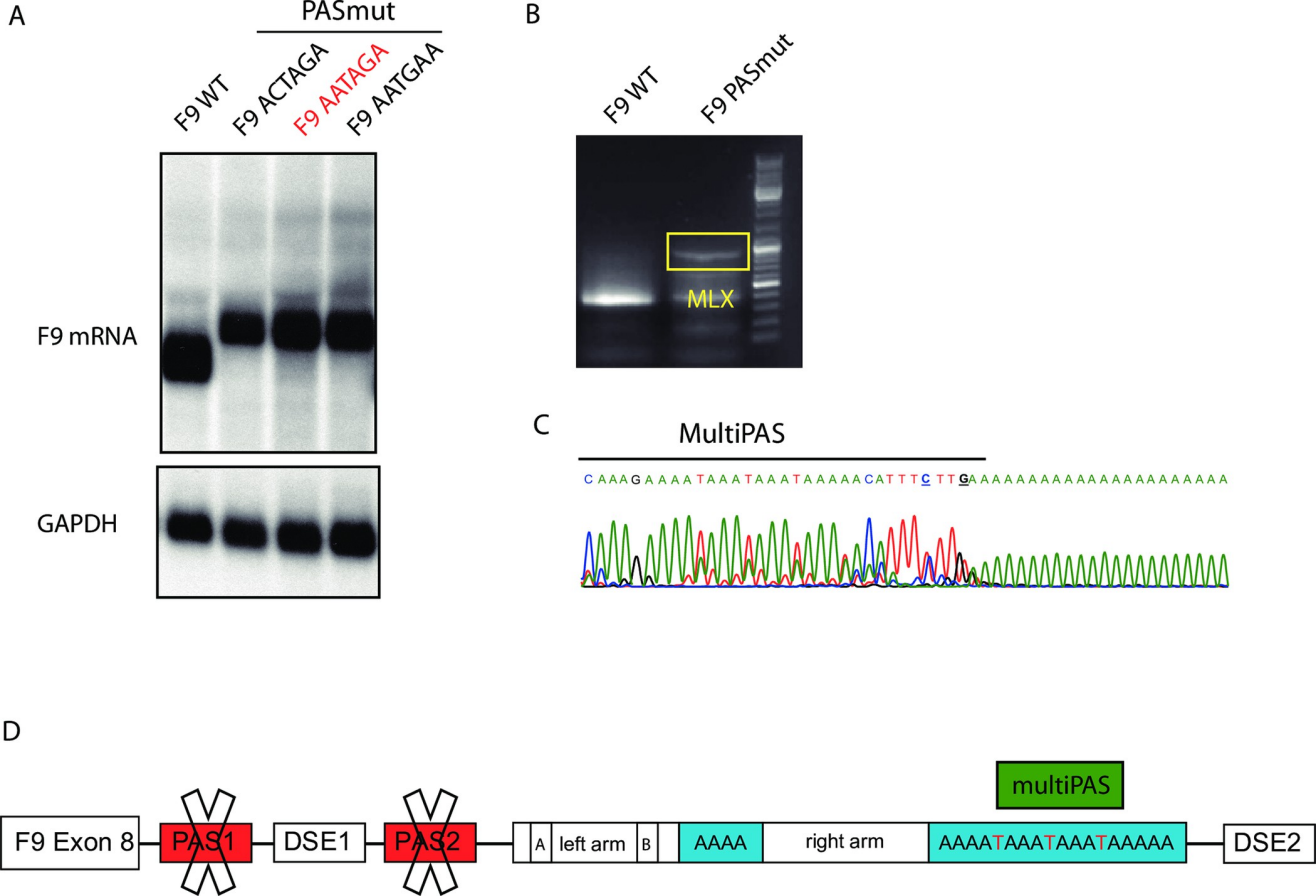
Modifications in the F9 PAS give rise to a longer mRNA species processed at a PAS. **A)** Northern blot of mRNAs collected from cells transfected with F9 minigenes holding various different PAS mutations. **B)** 3’ RACE performed on mRNAs obtained from HEK 293T cells upon transfection of F9wt and F9 PAS1 mutated minigenes. The yellow box highlights the band of a new F9 mRNA species polyadenylating at an alternative poly(A) site. **C)** Sanger sequencing results of the band highlighted in B). Sequencing indicates polyadenylation at a multi-poly(A) site located inside the Alu *Sx* element. **D)** Schematic showing a model of alternative polyadenylation regarding the non-functionality of PAS1 (due to mutation) and PAS2 due to a distanced downstream sequence element (DSE2). Polyadenylation therefore occurs at the multiPAS located at the 3’ end of the Alu *Sx* element.

To further characterize the mRNA derived from the PASmut minigene, we performed Northern blot analysis (Fig 2A). In addition, two F9 minigenes with alternative PAS1 modifications to ensure total polyadenylation inactivity (ACTAGA and AATGAA) were cloned and investigated. Intriguingly, a novel F9 mRNA species (~1 kb longer than the F9 wild type mRNA) was observed in all of the PAS1 variants (Fig 2A). This striking difference in mRNA length, however, does not match with an alternative polyadenylation event at PAS2, which already has been proved to be non-functional (Fig 2B). Using 3’RACE analysis and Sanger sequencing, we detected polyadenylation at the 3’ end of the Alu-element in case of all PAS1 variants (Fig 2B and 2C). The sequence analysis moreover revealed the presence of a triple AATAAA hexamer that we refer to as multiPAS. Taken together, these findings indicate the presence of a functional PAS inside the Alu *Sx* element (multiPAS) taking advantage of the DSE2 and a switch of polyadenylation upon inactivation of PAS1 and evidenced non-functionality of PAS2 as illustrated in Fig 2D.

### PAS2 regains its function after Alu-deletion

**Fig 3:**
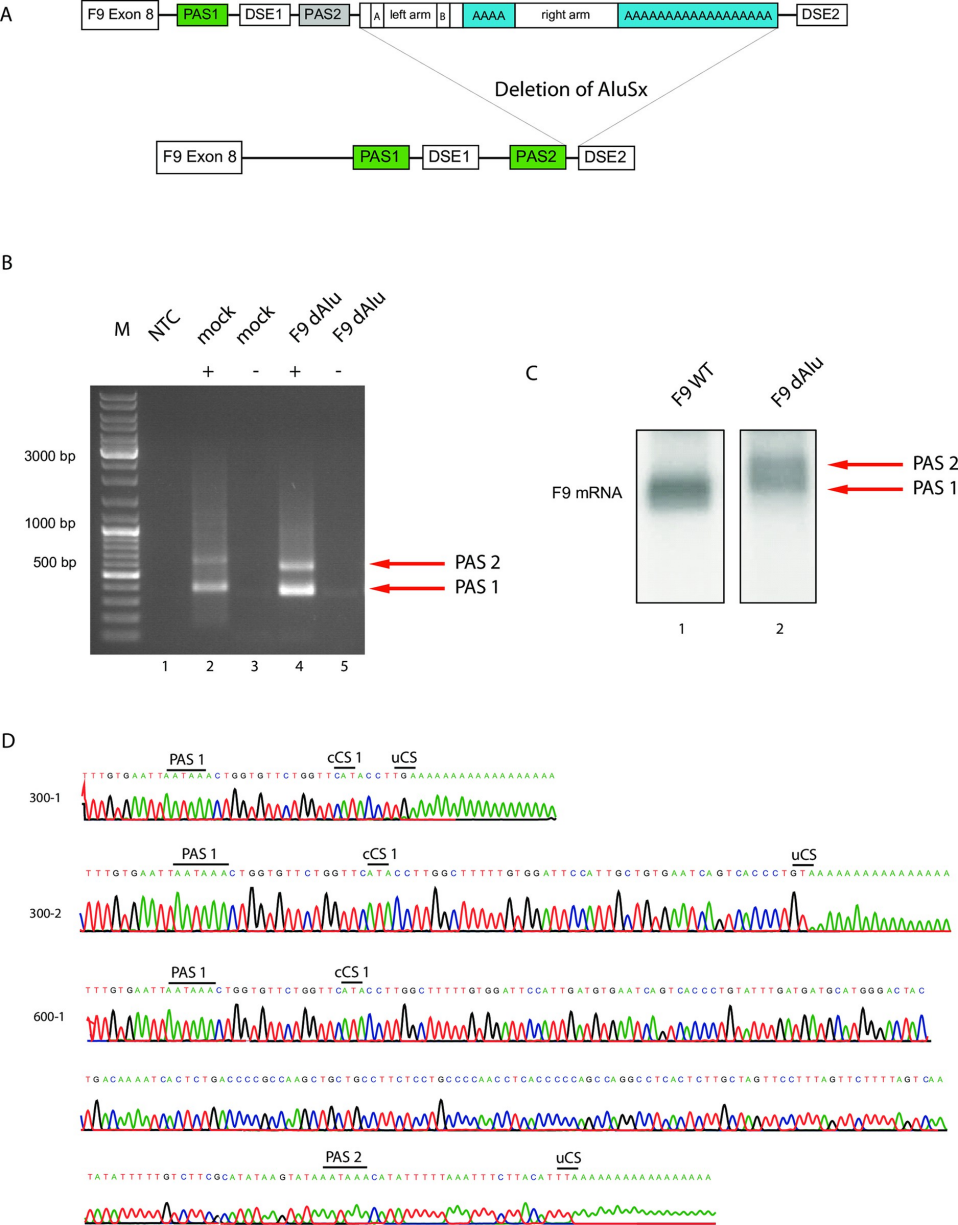
Deletion of Alu *Sx* element reactivates PAS2. **A)** Schematic depicting the arrangement of genomic elements upon deletion of the Alu *Sx* sequence. **B)** Gel electrophoresis of cDNA upon 3’RACE of mRNA of F9_wt_dAlu transfected HEK 293T cells. No template control (NTC) and mock serve as controls. +/- indicates the presence of reverse transcriptase inside the reaction. The bands in lane 2 indicate run slightly different and belong to the MLX gene (as validated by Sanger sequencing), which is aberrantly amplified by the F9-specific primers and also has a second polyadenylation site in its 3’UTR. Lane 4 shows two distinct lanes at 300 and 600 nts, which indicate polyadenylation at PAS1 and PAS2 of the F9 mRNA. **C)** Northern blot of mRNA obtained from HEK 293T cells transfected with F9_wt (lane 1) and F9_dAlu (lane 2). In contrast to the F9wt mRNA, the F9_dAlu mRNA exhibits two bands analogous to the 3’RACE and indicative of dual poly(A) site utilization. **D)** Sanger sequencing of the bands (300 bp or 600 bp) shown in lane 4. The sequencing results indicate polyadenylation at both PAS1 and PAS2, also with different cleavage sites (cCS = canonical cleavage site, uCS = unknown cleavage site).

To test the functionality of PAS2 prior to Alu invasion, we generated a F9 minigene deficient of the Alu element referred to as F9_wt_dAlu (Fig 3A). F9 mRNA analysis of HEK 293T cells transfected with this construct revealed utilization of both PAS1 and PAS2, as confirmed via 3’RACE and Northern blot (Fig 3B and 3C). The results of the 3’-RACE sequencing also indicate the utilization of different cleavage sites downstream of PAS1 (Fig 3D).

At this point we turned to species missing the Alu element and containing two functional PAS. This configuration applies to all canine species. A minigene combining human F9 cDNA and canine F9 3’UTR was generated (S2 Fig in S1 File) and polyadenylation was analyzed via 3’RACE. In this case, polyadenylation also at both canine PAS (cPAS) was observed.

These results support the assumption that PAS2 was functional in a common ancestor of primates and dogs long time before the invasion of Alu elements.

### A PAS1 mutation shortly after Alu *Sx* invasion would have resulted in a fatal FIX deficiency

**Fig 4:**
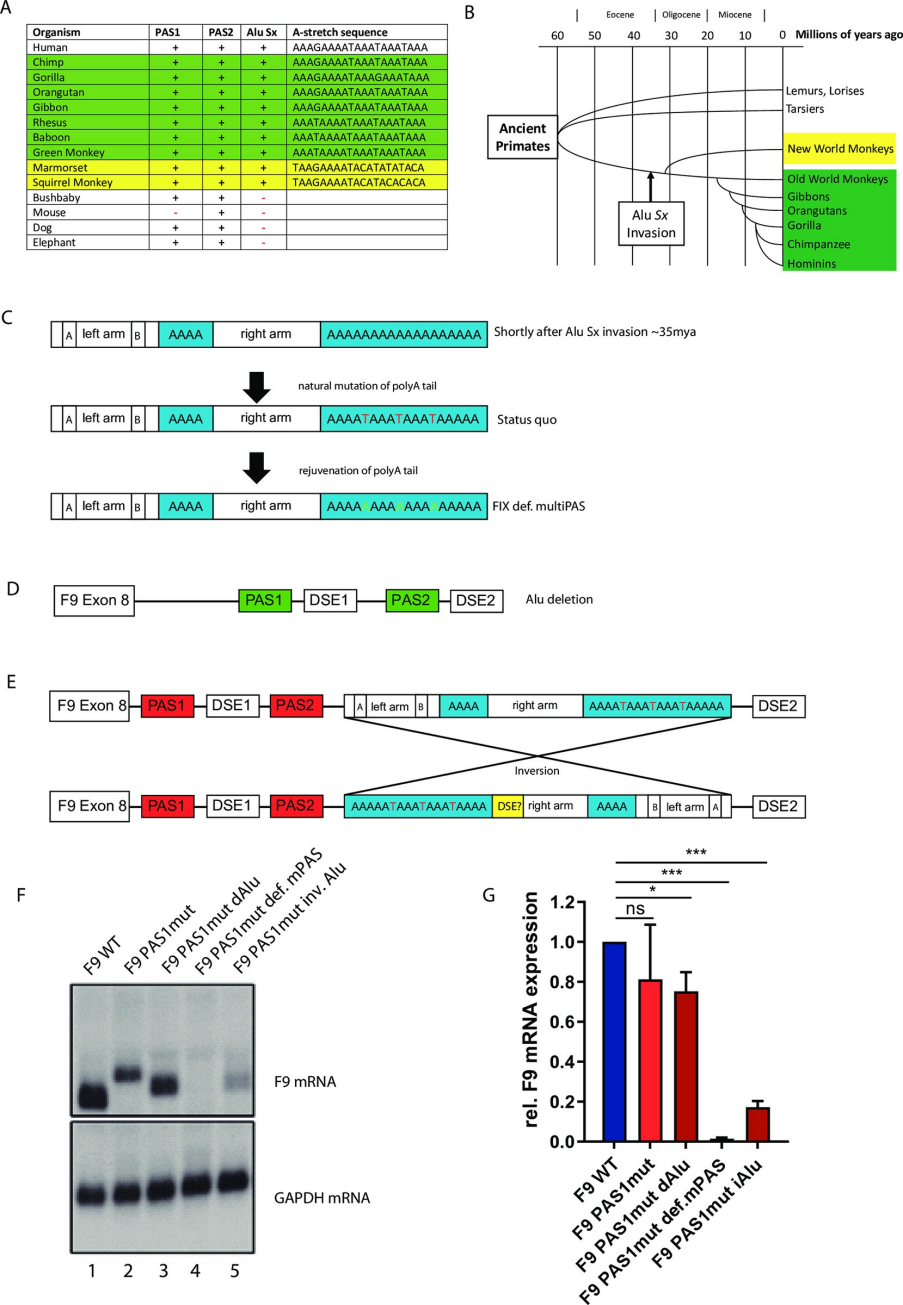
Comparison of poly(A) sites and terminal Alu *Sx* regions among different species. **A)** The sequence of the terminal Alu *Sx* element in several different species was compared in addition to the presence of a second poly(A) site (PAS1). Old World primates are highlighted in green, new world primates are highlighted in yellow. None of the New World primates holds a functional PAS in the Alu *Sx* element. **B)** Phylogenetic tree of primates over the last 60 million years and proposed Alu *Sx* invasion time point. The Alu *Sx* invasion must have occurred before separation of old world- and new world monkeys. However, only in old world monkeys a functional multiPAS is present. **C)** Schematic of a hypothetic Alu *Sx* element evolution. A pure poly(A) tail acquires T insertions over time (middle panel). By exchanging the T nucleotides with A nucleotides, the initial state of the poly(A) tail is restored (lower panel). The rejuvenated Alu *Sx* element is incorporated in the F9_def.multiPAS minigene. **D)** Schematic of a F9 minigene devoid of an Alu *Sx* element. **E)** Depiction of a minigene, which holds the Alu *Sx* element in an inversed orientation. This scenario should mimic the possibility of polyadenylation upon inverse Alu *Sx* insertion. **F)** Northern blot and **G**) results of the densitometric analysis upon phospho-imaging). HEK 293T cells were transfected with the respective minigenes and mRNA was subjected to Northern blot analysis (n = 3 biological replicates, error bars represent standard deviation. Student’s t test: *: p < 0.05 (exact p-value: 0.011); ***:p < 0.0001; ns: not significant. Exact p-values: 1).

The Alu *Sx* element downstream of the hF9 3’UTR is orientated in sense and has accumulated numerous mutations within its A-stretch giving rise to multiPAS complexes conserved in humans and the vast majority of old-world monkeys. Intriguingly, no PAS is abundant at the corresponding position in new-world monkeys according to the UCSC genome browser accessed in June 2023 (https://genome.ucsc.edu/ [29]) (Fig 4A and 4B). However, to investigate the effect of the PAS1 mutation shortly after invasion of the Alu *Sx* element into the primate genome approximately 30 mya (32 mya [30]), we rejuvenated its terminal A-stretch by reverting all A>T transversions that have accumulated over time (Fig 4C). This construct (F9_def. multiPAS) represents a complete null mutation in the patient context as no F9 mRNA could be detected upon transfection (Fig 4F, Lane 4). In a final scenario, we simulated the effect of the PAS1 mutation in the context of an inversely integrated Alu *Sx* element (Fig 4E), as it is known that they can insert in an antisense manner. A construct holding an inverse Alu *Sx* element at the exact same position intriguingly revealed a low mRNA expression. This residual expression of approximately 20% (compared to F9wt) emerges from polyadenylation at PAS2 and the inverted multiPAS (Fig 4E Lane 5), indicative of a cryptic DSE inside the inverted Alu *Sx* element.

### Comparative sequence analysis of the Alu *Sx* element in OWM and NWM reveals substantial differences

The stark differences in the multiPAS sequence between NWM (marmorset and squirrel monkey) and OWM evident in our initial genome browser analysis (Fig 4A) prompted us to substantiate this finding by analyzing more primate sequences. Based on a collection of primate genomes a multiple sequence alignment (MSA) of the terminal region of the F9 Alu *Sx* element was performed. Sequencing results of 546 OWM and NWM were aligned. The results of the MSA corroborate the observation made in the UCSC genome browser, which harbors the genomes of only 11 primate species (chimp, gorilla, orangutan, gibbon, rhesus, crab eating macaque, baboon, green monkey, squirrel monkey and bushbaby). Table 1, which only shows representative examples of the over 500 analyzed sequence, reveal a clear cut difference between both primate groups.

**Table 1:** Representative list of sequence motifs at the 3’ end of the F9 Alu Sx element.

**Species**	**Sequence Motif**	NWM
Cebus unicolor	-ATACATACATA	
Cebus albifrons	-ATACATACATA	
Cebus olivaceus	-ATACATACATA	
Chiropotes albinasus	-ATACATACATA	
Chiropotes satanas	-ATACATACATA	
Chiropotes sagulatus	-ATACATACATA	
Pithecia irrorata	-ATACATACATA	
Pithecia pithecia	-ATACATACATA	
Pithecia hirsuta	-ATACATACATA	
Pithecia monachus	-ATACATACATA	
Pithecia albicans	-ATACATACATA	
Cacajo calvus	-ATACATACATA	
Alouatta seniculus	-ATACATACATA	
Cercopithecus nictitans	AATAAATAAATA	OWM
Cercopithecus petaurista	AATAAATAAATA	
Cercopithecus pogonias	AATAAATAAATA	
Cercopithecus roloway	AATAAATAAATA	
Cercopithecus cephus	AATAAATAAATA	
Macaca leonina	AATAAATAAATA	
Hylobates lar	AATAAATAAATA	
Nomascus siki	AATAAATAAATA	
Semnopithecus priam	AATAAATAAATA	
Trachypithecus cristatus	AATAAATAAATA	

New World Monkeys shown in the upper panel of the table exhibit the motif “-ATACATACATA” at the position of the multiPAS “AATAAATAAATA” found in the Old World Monkeys. Sequencing data kindly provided by Prof. Dr. Tomas Marques, Universitat Pompeu Fabra, Barcelona.

Specifically, the MSA revealed the NWM-specific motif “-ATACATACATA” at the corresponding position of the multiPAS in OWM Table 1. These results clearly mark the separation of New World from Old World monkeys at this position in their genomes and therefore serves as a molecular clock landmark. Furthermore, the evolutionary path in NWM displays more or less random mutations that are acquired regularly by Alu poly A stretches. However, the selection of a multiPAS element may indicate some selective pressure. The nature of this selection is currently under investigation.

## Discussion

In our study, we have demonstrated the protective effect of an Alu *Sx* element on various mutations in the authentic PAS of the F9 gene. Initial analyses stated that polyadenylation of F9 mRNA exclusively occurs at PAS1. A second PAS (PAS2) located 223 nt downstream of PAS1, however, has been shown to be non-functional most likely due to the increased distance to its original DSE (DSE2), caused by the integration of the Alu *Sx* element ~35mya.

In the light of these findings, the question arose, why a patient with a predicted devastating poly(A) site mutation only exhibits a mild hemophilia B as reported by Li and colleagues[28].

To investigate this phenomenon, we have incorporated this particular mutation into a minigene holding the human F9 open reading frame, F9 3’ UTR and a downstream sequence including the Alu *Sx* element. Intriguingly, expression analyses via RT-qPCR indicated similar mRNA levels compared to the F9 wild type control (Fig 1D), despite the poor predicted polyadenylation activity of the mutated poly(A) site. On the level of coagulation activity, however, the PAS1 mutation has led to a certain but not massive reduction (Fig 1E), reflecting the mild disease severity described by Li and colleagues. This reduction is only partially due to an accelerated F9 mRNA decay (Fig 1G). We speculate that the extended 3’UTR terminating at the Alu element exhibit reduced translation efficiency. Along these lines, 3’UTR shortening was shown to correlate with higher protein output per mRNA molecule.

Further examination of the F9_PAS1mut mRNA via Northern blot revealed a novel mRNA species ~350 bp longer than F9_wild type mRNA (Fig 2A). 3’RACE analysis and Sanger sequencing evidenced a poly(A) switch into a multiPAS at the 3’ end of the Alu *Sx* element (Fig 2C).

Alu elements accumulate random mutations after a new insertion. The variant frequency reveals information on the time point of insertion, if compared to the parental sequence of that class of Alu elements. The multiPAS emerged from the terminal A-stretch of the Alu *Sx* element appears to take advantage of the DSE2, formerly associated with PAS2 as presented in Fig 3. Thereby, it facilitates alternative polyadenylation and near physiological mRNA expression levels in the case of the F9_PAS1 mutation in the patient. This exaptation of Alu elements is rare, but has been described before on a genome wide level [31,32]. This protection mechanism, however, only seems to be present in humans and old world primates.

Our finding, that the terminal A-stretch of the Alu *Sx* element contains multiple functional poly(A) sites, in turn raised the question of their formation. The generation of the PAS hexamer AATAAA from an A-stretch involves the event of a transversion (A>T), which occurs less common than a transition (A>G) as an interchange of purine and pyrimidine bases is required [33]. The constant disruption of the A-stretch with Ts in defined intervals and the thereby resulting poly(A) sites, infers the presence of a previous putative selective pressure towards polyadenylation at the Alu *Sx* element during evolution of Old world monkeys. In contrast, New world monkeys display no bias towards a multiPAS, but rather the default mutational drift after insertion of the Alu Sx.

Such a scenario in Old world monkeys could include a moderate mutational destruction of PAS1, still providing a basic coagulation activity but not a sufficient bleeding control upon blood loss (e.g. birth, injury). The generation of an alternative or additional poly(A) site inside the Alu Sx element by a single point mutation could have provided a significant selective advantage.

Although highly speculative at this point, parasites may also be considered as a potential selective pressure, which have acted on the formation of a multi-poly(A) site in the F9 3’UTR of all Old World monkeys. As malaria tropica caused by *Plasmodium falciparum*, which is endemic in West Africa until today, can induce disseminated intravascular coagulation (DIC), a modifying mechanism such as alternative polyadenylation as described above could have a beneficial effect on the survival of this disease and therefore led to the formation of a multiPAS. Assuming that the protective multiPAS has formed as a consequence of selective pressure, we aimed to investigate the effect of the PAS1 mutation shortly after insertion of the Alu *Sx* element into the F9 3’UTR. For this purpose, we have rejuvenated the A-stretch of the Alu *Sx* element by reverting all A>T mutations. Transfection of this construct (F9_PAS1mut_def.multiPAS) resulted in a complete loss of F9 mRNA expression (Fig 4F, Lane 4), indicative of the lethal effect of this mutation shortly after Alu *Sx* invasion, when no multiPAS was present. This again would support the above-mentioned hypothesis of a moderately impaired PAS1 prior to the multiPAS formation. Of course, the mutational bias in the homopolymeric A-stretch of the Alu Sx could also be explained by founder effects on populations passing through a genetic bottleneck. However, given the explanations above this seems unlikely.

To investigate the effect of the PAS1 mutation prior to Alu *Sx* invasion, we have generated a minigene deficient of it (F9_PASmut_dAlu). Our hypothesis that PAS2 functionality can be reconstituted upon spatial approximation of DSE2 via Alu *Sx* removal, proved to be correct, as a relative F9 mRNA expression level of approximately 70% was determined (Fig 4E, Lane 4). This result together with the finding that Alu deletion without PAS1-mutation leads to the utilization of both PAS1 and PAS2 (Fig 3) substantiates our model of PAS2 inactivation upon Alu *Sx* invasion. This poly(A) site constellation, however, precisely reflects the genomic situation of the bushbaby (Fig 4A), which constitutes the last primate without the Alu *Sx* element in the F9 3’UTR. In addition, both PAS1 and PAS2 appear to be conserved in *afrotheria* and *carniformia* species. Still, we were surprised that a perfect surgical removal of the Alu and thereby sending this genomic locus 38 mya back in time, reactivates PAS2 being silent and prone to mutations for such a long time.

In an additional scenario, we mimicked the antisense insertion of the Alu *Sx* element (Fig 4E). Interestingly, this modification has led to a residual F9 mRNA expression, which we explain with the presence of a putative DSE located inside the Alu *Sx* element. The antisense invasion of Alu elements close to a gene body and subsequent mutation of the A-stretch (A>T) to generate a PAS, which is even functional due to the presence of a DSE inside the Alu element, could be a potential mechanism of Alu elements to become vital and indispensable components of genes by generating novel transcripts that could be of advantage during a selection process.

Alu *Sx* elements are present in the genomes OWM and NWM. Strikingly, the multiPAS in the Alu *Sx* of the F9 gene is only conserved in OWM, but not in NWM (Fig 4A). NWM have separated approximately 40 mya [34]. Given that Alu *Sx* elements have invaded the primate genomes 35–55 mya, when Old World and New World have already separated geographically, the question arises, how this transposon has managed to integrate at identical positions in the genome of OWM and NWM simultaneously–on both sides of the Atlantic Ocean. As this scenario is rather unlikely, one explanation could be the transatlantic migration of primates into the New World shortly after Alu *Sx* invasion.

Our results indicate that NWM are not protected against a PAS1 mutation, which in turn implies the lack of an according selective pressure as possibly occurred in the OWM (e.g. a mild PAS1 mutation). In addition, the corresponding sequence motif in the Alu Sx element of the NWMs (ATACATACATA) was screened for RNA-binding factors using RBPmap [35]. However, no putative binding motif was detected, suggestive of the presence of a selective pressure on the preservation of the multiPAS in the Alu Sx element of the OWMs.

Based on the results of the MSA, we could show that especially the 3’ end of the Alu *Sx* element in the F9 3’UTR substantially differs between OWM and NWM. This specific difference may serve as an important molecular clock landmark for the separation event of OWM and NWM.

Taken together that Alu *Sx* invasion (30–50 mya) happened in all analyzed primates on the African continent except the Bushbaby, we assume that the separation of OWM and NWM must have occurred shortly after Alu invasion but shortly before a putative selective pressure event, which has led to the generation of the multiPAS in all OWMs.

By the time of OWM and NWM separation, Old World and New World were already ~2000 km apart, which raises the question of how and where exactly NWM have entered South America as there was no land bridge present and sea levels remained approximately 200 m higher than nowadays [36]. Accepted explanatory approaches for the transatlantic migration of primates consider “island hopping” and, particularly, the presence of “floating islands” [37–41]. Our finding that none of the analyzed NWMs holds a multiPAS sequence in the F9 Alu *Sx*, substantiates the assumption that most probably a single or at best a few transatlantic migration events of primates in the same period of time may have occurred indicative of a founder effect.

## Conclusion

In our study we highlight the beneficial effect of an Alu Sx element, which provides residual FIX activity in a hemophilia B patient by alternative polyadenylation in a Multi-PAS, which has evolved in the 3’ A-stretch. This specific feature is highly conserved in old world- but not in new world primates underlining the important role of this sequence in the transatlantic separation process of those species.

## Supporting information

S1 File (DOCX)
