# Supplementary material for: A transposable element prevents severe hemophilia B and provides insights into the evolution of new- and old world primates
Source: PLoS One. 2024 Oct 18;19(10):e0312303. doi: 10.1371/journal.pone.0312303 (PMC11488717; doi:10.1371/journal.pone.0312303)
Supplement: S1 File — (DOCX) [file pone.0312303.s001.docx]

# Supporting Information

**Figure S1**


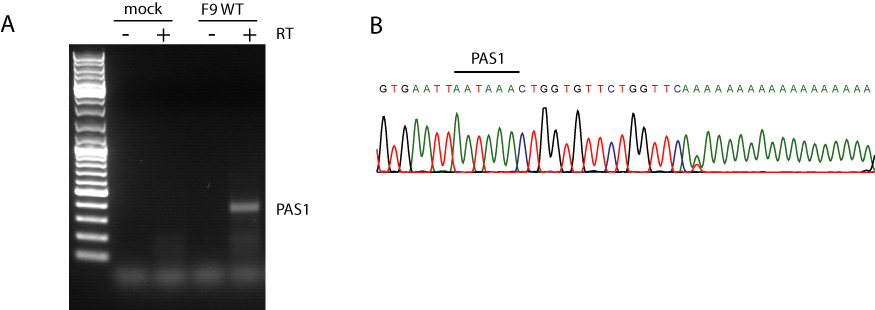


Figure S 1| **Polyadenylation of F9 mRNA.** **A)** Gel electrophoresis of a 3’RACE displaying the polyadenylation of the F9 mRNA upon transfection of the F9wt minigene into HEK 293T cells. Non-transfected cells (mock) serve as a control. Each condition was performed with (RT+) and without (RT-) reverse transcriptase added to the mRNA for subsequent cDNA synthesis to exclude DNA contamination. **B)** Sanger sequencing of the PAS1 band validating the usage of PAS1 for polyadenylation.

**Figure S2**


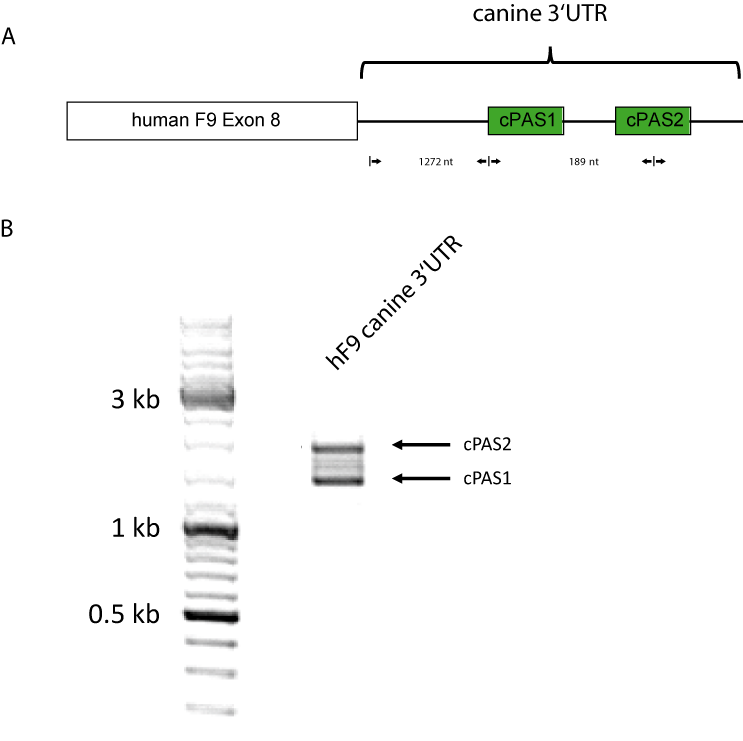


Figure S 2| **Generation of a minigene composed of the human F9 cDNA and the canine F9 3’UTR.** **A)** Schematic of the hF9 canine 3’UTR minigene, which holds two cPAS (canine PAS) in a distance of 189 nucleotides. The green color of cPAS1 and cPAS2 indicates their functionality. **B)** Gel electrophoresis of a 3’RACE performed on mRNA collected from cells, which were transfected with the hF9_canine 3’UTR minigene. The distance of the bands cPAS1 and cPAS2 correspond to their distance indicated in A.

**Figure S3_raw_images**


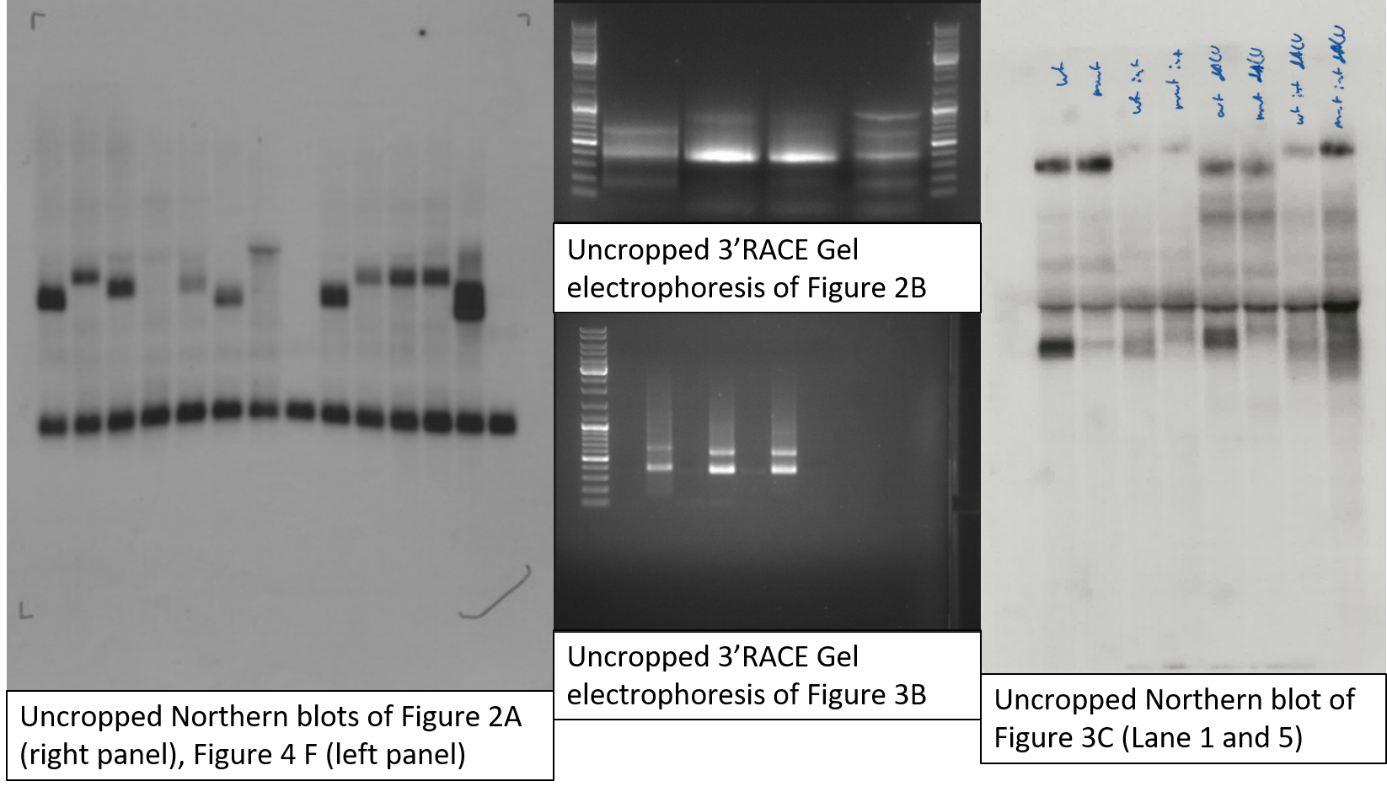


Figure S 3 | Uncropped and unmodified scans of Northern blots and Gel electrophoresis experiments depicted in the manuscript.

**Table S1**

| **Primer Name** | **Primer Sequence (5‘-3‘)** |
| --- | --- |
| P1 – PASmut1 fw | cttctagagagttgctgacaactgacgtatgttccctttgtgaatt**actcaa**ctggtgtt |
| P2 – PASmut2 fw | cttctagagagttgctgacaactgacgtatgttccctttgtgaatt**aatgaa**ctggtgtt |
| P3 – PASmut3 fw | cttctagagagttgctgacaactgacgtatgttccctttgtgaatt**aataga**ctggtgtt |
| P4 – PASmut rev | cagtcataagtgcggcgacg |
| P5 – dPAS_EcoRI_for | GAGGAAGAATTCAACAGTGTGTCTTCAGC |
| P6 – dAlu_OVL_us_rev | gaaacgcctcaagaaatgtaagaaattaaaaatatgtttatttatacttatatgcg |
| P7 – dAlu_OVL_ds_for | CATATTTTTAAATTTCTTACATTTCTTGAGGCGTTTCTTGTTAAATCATTC |
| P8 – dAlu_OVL_ds_rev | ggagaaaggcggacaggtatcc |
| P9 – F9 qPCR fw | ATTCCTATGAATGTTGGTGTCCCT |
| P10 – F9 qPCR rev | GGGTGCTTTGAGTGATG TTATCCAA |
| P11 – GAPDH qPCR fw | TTCACCACCATGGAGAAGGC |
| P12 – GAPDH qPCR rev | GGCATGGACTGTGGTCATGA |
| P13 – canine 3’UTR fw | GCGCCAATTGAGAAATTTGAAAAGACATTTATTGGT |
| P14 – canine 3’UTR rev | GCGCCAATTGAGAAATTTGAAAAGACATTTATTGGT |

Table S 1 | List of oligonucleotides used in this study.

**Table S2**

| *Raw data Figure 1E: F9 qRT-PCR values. Calculation was based on the 2- Δ Δct model upon normalization via GAPDH-Expression. Values normalized to F9 WT equal to 100%.* | | | | | | | |
| --- | --- | --- | --- | --- | --- | --- | --- |
|  |  |  |  |  |  |  |  |
|  | F9 WT | F9 PASmut |  |  |  |  |  |
|  | 1 | 0,972974 |  |  |  |  |  |
|  | 1 | 0,9058092 |  |  |  |  |  |
|  | 1 | 0,7707389 |  |  |  |  |  |
|  |  |  |  |  |  |  |  |
| *Raw data Figure 1E: F9 clotting activity raw data obtained by a one-step clotting assay.* | | | |  |  |  |  |
|  | F9 WT | F9 PASmut |  |  |  |  |  |
|  | 1 | 0,5769231 |  |  |  |  |  |
|  | 1 | 0,6923077 |  |  |  |  |  |
|  | 1 | 0,6923077 |  |  |  |  |  |
|  |  |  |  |  |  |  |  |
|  |  |  |  |  |  |  |  |
| *Raw data of Figure 1G: mRNA half-life measurement, values behind mean.* | | | | |  |  |  |
|  | hours | FIX wt |  |  | PAS1mut |  |  |
|  | 0 | 1 | 1 | 1 | 1 | 1 | 1 |
|  | 2 | 0,3830399 | 0,7804033 | 0,8591066 | 0,8043551 | 0,2116205 | 0,4550241 |
|  | 4 | 0,1827431 | 0,3737791 | 0,4143523 | 0,2506829 | 0,08016005 | 0,1275046 |
|  | 6 | 0,06148728 | 0,2063608 | 0,2014183 | 0,118933 | 0,0464588 | 0,05002609 |
|  | 8 | 0,03015448 | 0,1928775 | 0,1150212 | 0,05236381 | 0,01921599 | 0,0432347 |

| *Raw data of Figure 4F (Northern blot). Densitometric measurement of phosphor luminescence performed using Image Quant TL v2005.* | | | | | | |
| --- | --- | --- | --- | --- | --- | --- |
|  | FIX wt | FIX PAS1mut | FIX PAS1mut dAlu | FIX PAS1mut def mPAS | FIX PAS1mut inv. Alu |  |
|  | 1 | 0,8 | 0,78 | 0,02 | 0,21 |  |
|  | 1 | 1,09 | 0,85 | 0 | 0,18 |  |
|  | 1 | 0,55 | 0,68 | 0,01 |  |  |
